# Determinants of HIV testing and oral PrEP uptake among youth at Malawi University of Business and Applied Sciences: A cross-sectional study employing the Health Belief Model

**DOI:** 10.1371/journal.pgph.0006324

**Published:** 2026-06-24

**Authors:** Josephine Katharina Mommertz, Martha Kamanga, Wongani Mtabayira Nyondo, Patrick Mapulanga, Lily Caroline Kumbani

**Affiliations:** 1 Reproductive Health Department, School of Neonatal, Maternal and Reproductive Health, Blantyre, Malawi; 2 Midwifery Department, School of Neonatal, Maternal and Reproductive Health, Blantyre, Malawi; 3 Library Department, Kamuzu University of Health and Sciences, Lilongwe, Malawi; PLOS: Public Library of Science, UNITED STATES OF AMERICA

## Abstract

In Malawi, approximately 9000 new HIV infections occur annually among youth. As of 2017, only 50.2% of young men and 68.2% of young women aged 15–24 years had been tested for HIV infection. Oral pre-exposure prophylaxis is recommended for HIV-seronegative at-risk individuals and has been available to youth since 2022. This study assessed the determinants of HIV testing and oral PrEP uptake among youth at the Malawi University of Business and Applied Sciences, guided by the Health Belief Model. A cross-sectional study was conducted using a stratified random sampling method to select 377 youth aged 18–24 years. Data were collected using a self-administered questionnaire and analyzed using STATA software version 14.1, with statistical significance set at p < 0.05. Descriptive statistics and multivariable logistic regression analyses were performed. One factor was associated with HIV testing: being aged 21–24 years (AOR = 2.5; 95%CI = 1.34,4.59). Seven factors were significantly associated with oral PrEP uptake; being male (AOR = 2.8; 95%CI = 1.23,6.72), perceiving a threat of HIV infection (AOR = 5.7; 95%CI = 1.80,18.18), testing for HIV at a three-month interval (AOR = 5.5; 95%CI = 1.92,15.52), knowing the partner’s HIV status (AOR = 2.5; 95%CI = 1.04,5.86), knowing someone taking oral PrEP (AOR = 4.8; 95%CI = 2.17,10.71), having awareness about PrEP (AOR = 11.4; 95%CI = 1.08,119.90), and being willing to take a daily pill (AOR = 1.9; 95%CI = 0.84,4.09). External cues showed that youth primarily obtained information about HIV testing and oral PrEP from social media and peers. In conclusion, associations highlighted the importance of involving partners and peers in promoting PrEP uptake. Digital media served as an important source of information among youth, and targeted health communication strategies could leverage these channels as effective cues to increase HIV testing and oral PrEP uptake.

## Introduction

Malawi remains one of the countries most affected by human immunodeficiency virus (HIV) in sub-Saharan Africa (SSA). An estimated one million people in Malawi are currently living with HIV, with a national adult prevalence among individuals 15 years and above of 8.9% [1]. Youth aged 15–24 years represent a key priority population, accounting for a substantial proportion (36%) of all HIV infections in the country [1]. The annual HIV incidence among youth (0.25%) is slightly higher than the overall adult incidence, resulting in approximately 9,000 new infections annually. In Blantyre city, where this study was conducted, HIV prevalence reaches 14.2%, exceeding the national average [1]. Persistent new infections among youth are associated with early sexual debut, low condom use, multiple sexual partners and substance use [2,3].

In response to the epidemic, Malawi’s National AIDS Commission implemented the Universal Test and Treat (UTT) policy [4] and introduced oral pre-exposure prophylaxis (PrEP) in 2018 as an additional prevention strategy [5]. Oral PrEP is a daily medication designed to protect HIV-negative individuals in high-risk populations by significantly reducing the risk of infection by up to 90%, when taken consistently [6,7]. The Ministry of Health (MoH) has prioritized youth aged 15–24 years for PrEP access and identified universities as strategic platforms for delivering both HIV testing services (HTS) and oral PrEP given the high concentration of youth within these settings [5].

Despite these national efforts, critical gaps remain in the uptake of HTS and oral PrEP among youth. In 2017, 50.2% of young men and 68.2% of young women aged 15–24 years reported ever having been tested for HIV [8]. Uptake of oral PrEP has progressed more slowly than anticipated, with evidence from the region indicating similarly low levels of utilization [9,10]. University settings may represent high-risk environments due to increased autonomy, peer influence, and engagement in high-risk sexual behaviors [11–13]. Although campus health clinics and youth-focused initiatives have been established, barriers such as limited awareness, stigma, low perceived susceptibility to HIV infection, and reluctance to initiate daily medication continue to impede service uptake [14–16]. Evidence on service utilization within university settings remains limited, and the psychosocial determinants influencing uptake are not well understood.

The Health Belief Model (HBM) provides a theoretical framework to examine how perceived susceptibility, perceived benefits, perceived barriers and cues to action shape prevention behaviors [17]. In this study, selected HBM constructs are applied to explore psychosocial factors associated with prevention service utilization within the university context. Unlike previous studies that have examined these services separately, this study analyzes both outcomes within a single analytic framework. Therefore, this study aims to assess the determinants of HIV testing and oral PrEP uptake among university students in Blantyre, Malawi, using the HBM framework.

## Methods

### Ethics statement

This study was conducted in accordance with the Declaration of Helsinki and received ethical approval from the College of Medicine Research and Ethics Committee of Kamuzu University of Health Sciences (approval number P.11/23–0447). Administrative clearance to conduct the study was obtained from MUBAS.

Before completing the questionnaire, the study purpose and procedures were explained verbally and through a written information sheet. Respondents were informed of the voluntary nature of the study, their right to withdraw at any time, and the measures taken to ensure confidentiality and anonymity. Written informed consent was obtained from all respondents aged 18 years and above. Questionnaires were completed in a private setting to ensure privacy. Respondents received a small compensation for the time spent completing the questionnaire.

### Study design

This study employed a descriptive cross-sectional design. The study was guided by selected constructs of the HBM, a widely used framework for understanding health-related behaviors. The HBM informed the development of the questionnaire and the selection of variables included in the analysis.

In this study three constructs of the HBM were operationalized: modifying factors, perceived threat and cues to action. Modifying factors included selected demographic and socio-cultural characteristics that may indirectly influence health behavior by shaping perceptions of risk. Perceived threat comprised perceived susceptibility to HIV infection and perceived severity of its health and social consequences [18]. Cues to action referred to external or internal prompts that may stimulate preventive behavior [18]. In the university context, cues encompassed awareness and availability of HIV testing and oral PrEP services on campus, such as peer mobilization, informational posters, and the services provided by the campus health clinic.

While the HBM includes additional constructs, such as perceived benefits and perceived barriers, and self-efficacy [18], this study focused on the three constructs feasible within the scope of the survey.

Two binary outcomes were assessed (ever tested for HIV and ever used oral PrEP). The HBM provided the conceptual structure for organizing independent variables and guiding analytical approaches.

### Study setting

This study was conducted at Malawi University of Business and Applied Sciences (MUBAS) in Blantyre, Malawi’s second-largest city, in February 2024. MUBAS, a non-medical public university, was selected due to the availability of on-campus preventive HIV services and its relevance to examine PrEP uptake and HIV testing among youth. In line with national guidelines, the campus Health Clinic offers youth-friendly services for enrolled students, including voluntary HIV testing (rapid tests), HIV self-testing kits, sexually transmitted infection (STI) screening, and same-day oral PrEP initiation. Services are free and accessible via appointment or walk-in. Oral PrEP has been available since January 2022, and HIV testing serves as the entry point for PrEP initiation without the need for external referral. The on-campus availability of these services facilitates access to preventive care.

Malawi’s HTS framework aims to reduce HIV/AIDS impact and eliminate it as a public health threat by 2030 [4]. Within this framework, the 2016 UTT policy promotes routine and voluntary HIV testing at all health facility entry points with immediate initiation of antiretroviral therapy (ART) for individuals testing HIV-positive [4]. This approach has been complemented by the national scale-up of HIV-self testing. Eligibility for oral PrEP initiation is based on standardized risk assessment conducted by a health professional, including age, HIV status and relevant medical history [19]. PrEP can be initiated on the same day following a negative HIV test result with follow-up HIV testing after one month and subsequently every three months, in line with national guidelines [20]. These national policies are reflected in the services provided at MUBAS campus clinic.

Despite the availability of these services, uptake remains low. As of March 14th, 2024, 104 students were actively using oral PrEP (17 young women and 87 young men), representing a small proportion of the student population. Informal input from clinic staff indicates that challenges among university students include low perceived risk, academic workload, and occasional sharing of PrEP medication. To strengthen uptake, 12 PrEP ambassadors mobilize peers for PrEP uptake, support PrEP refills, and promote adherence. Posters, follow-up calls and outreach during university events further facilitate engagement with PrEP services.

### Recruitment of study respondents

**Fig 1:**
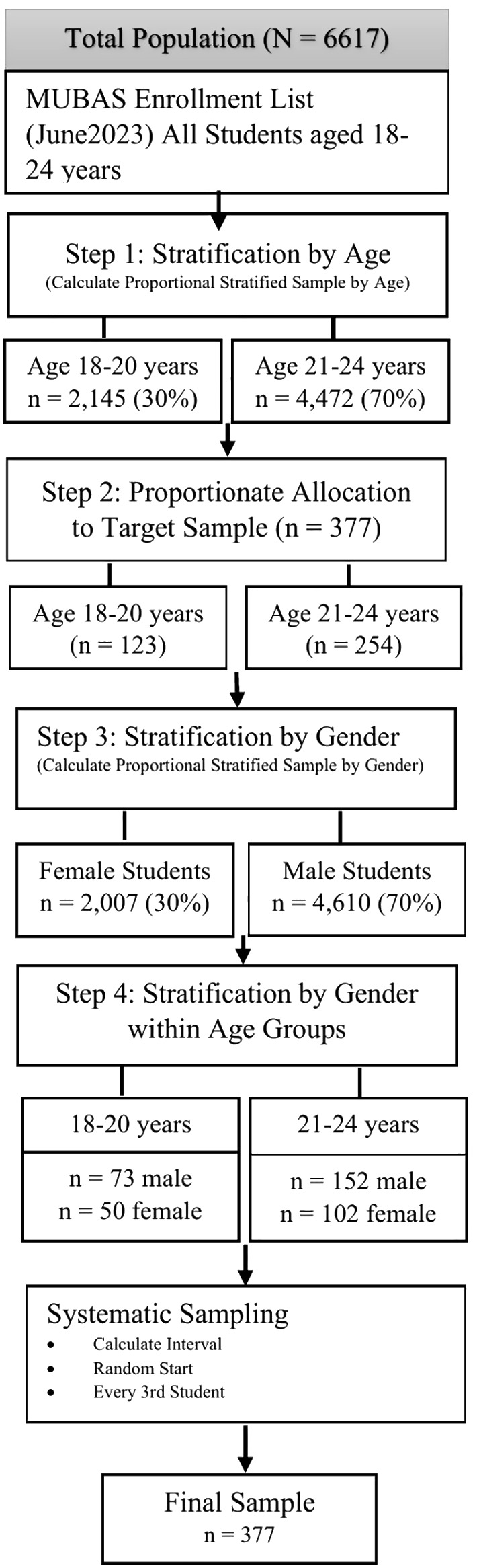
Sampling method flowchart.

The study population was limited to youth aged 18–24 years enrolled at MUBAS. Only respondents who were registered at MUBAS and autonomous in providing information, as well as answering the questionnaire were included. Student status was verified by requesting respondents to present a valid MUBAS student ID card before completing the survey. Respondents with special learning needs requiring additional support were excluded from the study (Fig 1).

### Sample size determination

Yamane’s formula was used for the sample size calculation [21]:


$$\begin{array}{c} n=\;\frac{N}{\text{1}+N{\left(e\right)}^{\text{2}}} \end{array}$$

where n is the expected sample size, N is the population of the study, and e is the margin of error (5% [0.05] if the confidence interval is 95%). The sample size was calculated as follows:


$$\begin{array}{c} n=\;\frac{\text{6}\text{6}\text{1}\text{7}}{\text{1}+\text{6}\text{6}\text{1}\text{7}{\left(\text{0}.\text{0}\text{5}\right)}^{\text{2}}}\;,\;n\;=\;\text{3}\text{7}\text{7} \end{array}$$

### Sampling method

#### Data collection procedure

A self-administered questionnaire was used to collect data from the study population (S1 File). Questionnaires were distributed to the study respondents, and 20 minutes were required to complete the questionnaire. Data were collected in a private room of the university library. Two research assistants were involved in data collection under the supervision of the principal investigator. They received training on study procedures, including obtaining informed consent, ensuring ethical principles, clarifying instructions without leading responses, and checking returned forms for completeness.

#### Data collection instrumentation

A structured questionnaire was developed using validated items from the 2015–2016 Malawi Demographic and Health Survey and from previously published tools [22–25]. The tool was written in English and combined closed-ended and multiple-choice questions. English was used because it is the language of instruction from fifth grade onwards in Malawi [26].

The questionnaire was divided into six sections (A to F), with a total of 26 questions. The first three sections (A-C) captured modifying factors as defined in the HBM. Section A on sociodemographic information comprised six questions with multiple responses options. Section B included two questions on youths’ drinking patterns, with one closed-ended and one multiple-choice formats. Section C contained six questions on sexual activity, using both closed and multiple-choice formats. Section D assessed risk perception using two self-reported items, both reflecting respondents’ subjective evaluation of their likelihood of HIV infection. Section E addressed HIV testing behavior and collected information on the source from which respondents obtained information about testing. Section F assessed oral PrEP use and collected information on the sources from which respondents obtained information about PrEP.

#### Measures

##### Dependent variables.

This study included two primary dependent variables: HIV testing and the uptake of oral PrEP.

HIV testing was measured using the question “Have you ever been tested for HIV?” with binary response options: “Tested” or “Not tested”. Oral PrEP uptake was assessed by the question “Have you ever used oral PrEP?” with binary options: “Taking oral PrEP” or “Not taking oral PrEP”.

##### Independent variables.

Within the HBM, modifying factors are conceptualized as contextual characteristics that indirectly influence health behavior by shaping risk perceptions. In this study, these factors were examined as independent variables to assess their direct associations with HIV testing and oral PrEP uptake, acknowledging that this differs from the model’s traditional conceptualization. Modifying factors included sociodemographic factors (gender, age, educational level, ethnicity, relationship/marital status) and socio-cultural behavioral factors (sexuality, alcohol consumption, alcohol quantity, age at first sex, condom use first sex, condom use last sex, frequency of condom use, sexual partners’ age, lifetime partners, no condom use at least once, and willingness to take pill daily).

#### Validity and reliability

##### Validity.

The face and content validity of the questionnaire were assessed prior to data collection. The questionnaire was developed using previously validated items and was reviewed by experts to evaluate face and content validity. Experts in HIV/AIDS and reproductive health assessed whether the items adequately captured the intended constructs. Youth specialists reviewed the questionnaire for content validity, focusing on relevance, clarity, and appropriateness for the target age group. Feedback from both expert groups informed revisions to item wording and structure to enhance comprehensibility. To enhance external validity, a combination of stratified and systematic random sampling was used to obtain a representative sample.

##### Reliability.

The questionnaire primarily consisted of single-item measures, such as socio-demographics, alcohol consume, sexual activity, risk perception, HIV testing, and PrEP uptake. As these items measure distinct constructs and are not combined into multi-scales, conventional reliability statistics were not applicable. Reliability was ensured through a pre-test conducted on 2 February 2024 among 11 MUBAS students to assess clarity, flow, and usability. Feedback from pre-test was used to refine unclear items. research assistants received additional training in the informed consent process, questionnaire administration, and completeness checking. All completed questionnaires were reviewed on-site, and any missing or unclear responses were clarified with the participants when possible.

#### Data analysis

Data were analyzed using STATA version 14.1. Descriptive statistics summarized participants’ characteristics and cues to action, presented as frequencies and percentages. The study assessed associations with HIV testing and oral PrEP uptake, both defined as binary outcomes (yes/no). Logistic regression was therefore selected as a method for modeling associations between predictor variables and binary outcomes. While alternative approaches, such as modified Poisson regression, may provide more directly interpretable estimates for common outcomes, logistic regression was preferred due to its widespread use and suitability for modeling binary outcomes. HBM-related modifying factors and perceived threat were included as independent variables. Variables with a p-value <0.05 in bivariate analysis alongside HBM constructs (PrEP awareness, knowledge of the partner’s HIV status, knowing someone taking PrEP and HIV concerned) were included in multivariable logistic regression. Results are presented as crude odds ratios (COR), adjusted odds ratios (AOR), 95% confidence intervals, and p-values.

## Results

### Sociodemographic characteristics of respondents

The majority of the respondents (58.9%, n = 222) were males; most (65.6%, n = 247) were 21–24 years (mean age 21; SD 1.7); the majority (95.6%, n = 356) were undergraduates, and most (75.9%, n = 286) were single (Table 1).

**Table 1:** Sociodemographic characteristics of youth at MUBAS, Malawi (N = 377).

Variables	n	Percentage (%)
**Gender**		
Female	155	41.1
Male	222	58.9
**Age (group)**		
18-20 years	130	34.4
21-24 years	247	65.6
**Ethnicity** (n = 335)		
Chewa	79	23.6
Lomwe	74	22.1
Tumbuka	68	20.3
Ngoni	64	19.1
Others	50	14.9
**Educational Level** (n = 372)		
Undergraduate	356	95.6
Postgraduate	8	2.2
Diploma	8	2.2
**Relationship/Marital status**		
Single	286	75.9
Casual dating	63	15.8
Having a partner	21	5.6
Married	6	1.6
Other	1	0.3

### Sexual-risk behavior characteristics of respondents

A substantial proportion of respondents (33.2%, n = 125) reported consuming alcohol, with varying drinking patterns (Table 2). The majority (67.4%, n = 254) were sexually active, and most (64.5%, n = 158) had their sexual debut between the ages of 15–19 years (Table 2). The median age at sexual debut was 18 years (IQR: 18–20). While 71.9% (n = 179) used a condom during their last sexual encounter, consistent use was reported by only 49.4% (n = 123) (Table 2). Most respondents (59.1%, n = 136) had 1–2 lifetime sexual partners, defined as the number of individuals they had ever had sexual intercourse with. Across the full sample, the mean number of lifetime partners was 3 (SD = 2) (Table 2).

**Table 2:** Sexual-risk behavior among youth at MUBAS, Malawi (N = 377).

Variable	n	Percentage (%)
**Alcohol consumption** (n = 377)		
Yes	125	33.2
No	252	66.8
**Alcohol quantity** (n = 125)		
Occasional	78	62.4
Moderate	41	32.8
Frequent	6	4.8
**Ever had sex** (n = 368)		
Yes	254	67.4
No	114	30
**Sexual Behavior** (n = 254)		
Heterosexual	247	97.2
Men who have sex with men	4	1.6
Women who have sex with women	2	0.8
Sex with both	1	0.4
**Age (group) at first sex** (n = 245)		
10-14 years	9	3.7
15-19 years	158	64.5
20-24 years	78	31.8
**Condom use of last sex** (n = 249)		
No	70	28.1
Yes	179	71.9
**Frequency of condom use** (n = 249)		
Always	123	49.4
Sometimes	106	42.6
Never	20	8
**Condom use at least once** (n = 251)		
No	116	46.2
Yes	135	53.8
**Number of lifetime partners** (n = 230)		
1-2 partners	136	59.1
3-5 partners	74	32.2
6-9 partners	13	5.7
10 or more partners	7	3
**Age (group) in years of the sexual partner** (n = 212)		
15-18 years	27	12.7
19-24 years	142	67
25-29 years	39	18.4
30 years and above	4	1.9
**Knowledge of the partner’s HIV status** (n = 291)		
No	132	45.4
Yes	159	54.6
**Perceived susceptibility to HIV/AIDS of STI transmission** (n = 371)		
No	258	69.5
Yes	113	30.5
**Perceived threat of HIV** (n = 370)		
Not really	111	30
Sometimes	76	20.5
Yes	183	49.5

### HIV testing and oral PrEP uptake characteristics of respondents

The majority (72.8%, n = 271) had ever been tested for HIV. Among those tested, 58.7% (n = 159) were male and 41.3% (n = 112) were female, all aged 18–24 years (Table 3). Among these, most (42.8%, n = 126) had their most recent HIV test occurring two or more years ago (Table 3). Most respondents (85.1%, n = 320) were aware of oral PrEP, and 65.1% (n = 239) expressed willingness to take a daily pill to prevent HIV infection (Table 3). Actual oral PrEP uptake was 21.7% (n = 82), with the majority obtaining the prophylaxis from the MUBAS Health Clinic (78%, n = 64) (Table 3). Most PrEP users were male respondents aged 18–24 years (75.6%, n = 62) (Table 3).

**Table 3:** HIV testing and oral PrEP characteristics among youth at MUBAS, Malawi (N = 377).

Variables	n	Percentage (%)
**Ever been tested for HIV** (n = 372)		
Yes	271	72.8
No	101	27.2
**Ever been tested by gender** (n = 271)		
Female	112	41.3
Male	159	58.7
**Most recent HIV test** (n = 294)		
1-3 months	72	24.5
4-6 months	52	17.7
7-11 months	29	9.9
12-23 months	15	5.1
2 or more years	126	42.8
**Three-month testing interval** (n = 363)		
Yes	46	12.7
No	317	87.3
**PrEP awareness** (n = 376)		
Yes	320	85.1
No	56	14.9
**Ever accessed PrEP** (n = 377)		
Yes	82	21.7
No	295	78.3
**PrEP distribution by facility** (n = 82)		
MUBAS^a^ Health Clinic	64	78
QECH^b^	9	11
Lighthouse Clinic	4	5
Other	5	6
**PrEP access by gender** (n = 82)		
Female	20	24.4
Male	62	75.6
**Willingness to take pill daily** (n = 367)		
Yes	239	65.1
No	128	34.9

^a^ MUBAS = Malawi University of Business and Applied Sciences

^b^ QECH = Queen Elizabeth Central Hospital

### Determinants of HIV testing among youth at MUBAS

Multivariable logistic regression analysis showed that two factors were significantly associated with HIV testing. Respondents aged 21–24 years had more odds to be tested for HIV than those aged 18–20 years (AOR = 2.5, 95%CI = 1.34,4.59, p-value = 0.004) (Table 4). The odds of HIV testing had higher odds among respondents who reported testing every three months than among those who did not test regularly (AOR = 11.4, 95%CI = 1.49,86.64, p-value = 0.019) (Table 4).

**Table 4:** Determinants of HIV testing among youth at MUBAS, Malawi.

Variable	COR^a^	p-value	95% CI	AOR^b^	p-value	95% CI
**Modifying factors**						
**Age**						
18-20	Ref			Ref		
21-24	2.6	<0.001*	[1.61,4.12]	2.5	0.004*^c^	[1.34,4.59]
***Perceived threat***						
No	Ref			Ref		
Yes	1.2	0.161	[0.94,1.48]	1.4	0.298	[0.73,2.74]
***Knowledge of the partner’s HIV status***						
No	Ref			Ref		
Yes	2.5	0.002*	[1.39,4.31]	1.8	0.068	[0.96,3.31]
***Three-month HIV testing interval***						
No	Ref			Ref		
Yes	17.3	0.005*	[2.35,127.68]	11.4	0.019*^c^	[1.49,86.64]
***Awareness about PrEP***						
No	Ref			Ref		
Yes	1.8	0.058	[0.98,3.32]	1.5	0.410	[0.60,3.53]
***Knowing someone taking Prep***						
No	Ref			Ref		
Yes	1.4	0.177	[1.94,5.37]	0.8	0.548	[0.42,1.59]

^a^ COR means Crude Odds Ratio.

^b^ AOR means Adjusted Odds Ratio.

^c^ [*] Significant at 5% significance level.

### Model diagnostic

From the goodness of fit test to test the fitting of the model, a p value of 0.850 was obtained. This shows that the model does fit well as the null hypothesis of “model fits well” is failed to be rejected at 95% confidence level (Chi square = 125.59, p-value = 0.8500). Considering the R-Squared, 10.2% of the changes in HIV testing is attributed by the factors in the model (R squared = 0.102).

### Determinants of oral PrEP uptake among youth at MUBAS

Multivariable logistic regression identified seven factors significantly associated with oral PrEP uptake. Young men had higher odds to take oral PrEP than young women (AOR = 2.8, 95%CI = 1.23,6.72, p-value = 0.015) (Table 5). Respondents who perceived HIV infection as a threat had higher odds to access oral PrEP than those who did not (AOR = 5.7, 95%CI = 1.80,18.18, p-value = 0.003) (Table 5). The odds of oral PrEP uptake were also higher among respondents who were tested for HIV every three months than among those who did not test regularly (AOR = 5.5, 95%CI = 1.92,15.52, p-value = 0.001) (Table 5). Respondents who knew their partner’s HIV status had higher odds of taking oral PrEP than those who were unaware (AOR = 2.5, 95%CI = 1.04,5.86, p-value = 0.042) (Table 5). Respondents who were aware of oral PrEP had higher odds to use it than those who were unaware (AOR = 11.4, 95%CI = 1.08,119.90, p-value = 0.043) (Table 5). Respondents who knew someone taking oral PrEP had higher odds to use it themselves (AOR = 4.8, 95%CI = 2.17,10.71, p-value<0.001) (Table 5). Respondents who were willing to take a daily pill had higher odds to take oral PrEP (AOR = 1.9, 95%CI = 0.84,4.09, p-value<0.001) (Table 5).

**Table 5:** Determinants of oral PrEP among youth at MUBAS, Malawi.

Variable	COR	p-value	95% CI	AOR	p-value	95% CI
**Modifying factors**						
***Gender***						
Female	Ref			Ref		
Males	2.6	0.001*	[1.46, 4.44]	2.8	0.015^*^	[1.23,6.72]
***Perceived threat of HIV***						
No	Ref			Ref		
Yes	2.2	0.012*	[1.19,4.05]	5.7	0.003*	[1.80,18.18]
***Three-month HIV testing interval***						
No	Ref			Ref		
Yes	3.1	0.001*	[1.61,5.99]	5.5	0.001*	[1.92,15.52]
***Number of lifetime sexual partners***						
1-2	Ref			Ref		
3-5	1.8	0.084	[0.93,3.33]	0.8	0.563	[0.35,1.78]
6-9	5.7	0.004*	[1.74,18.88]	4.2	0.065	[0.91,19.62]
10+	4.8	0.048*	[1.01,22.59]	2.7	0.379	[0.29,26.40]
***Knowledge of the partner’s HIV status***						
No	Ref			Ref		
Yes	2.1	0.006*	[1.24,3.70]	2.5	0.042*	[1.04,5.86]
***Awareness about PrEP***						
No	Ref			Ref		
Yes	8.6	0.003*	[2.06,36.32]	11.4	0.043*	[1.08,119.90]
***Knowing someone taking Prep***						
No	Ref			Ref		
Yes	3.2	<0.001*	[1.94,5.37]	4.8	<0.001*	[2.17,10.71]
***Willingness to take a pill daily***						
No	Ref			Ref		
Yes	2.4	0.004*	[1.31,4.23]	1.9	<0.001*	[0.84,4.09]

### Model Diagnostic

From the goodness of fit test to test the fitting of the model, a p value of 0.1973 was obtained. This shows that the model does fit well as the null hypothesis of “model fits well” was failed to be rejected at 95% confidence level (Chi square = 87.33, p-value = 0.1973). Considering the R-Squared, 26.9% of the changes in prep uptake are attributed by the factors in the model (R squared = 0.269).

### Cues to action for HIV testing and oral PrEP uptake among youth at MUBAS

**Fig 2:**
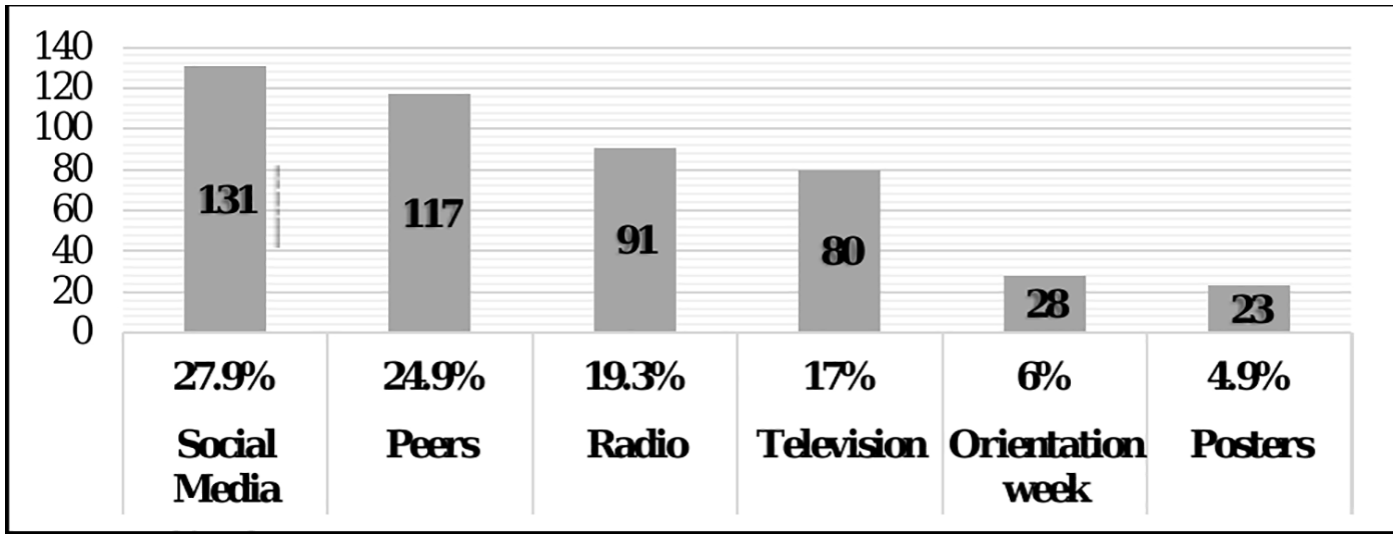
Sources of information on HIV testing affecting youth at MUBAS, Malawi.

The majority of respondents (27.9%, n = 131) received their information from social media, while only 6% (n = 28) reported the MUBAS orientation week as their source of information (Fig 2).

**Fig 3:**
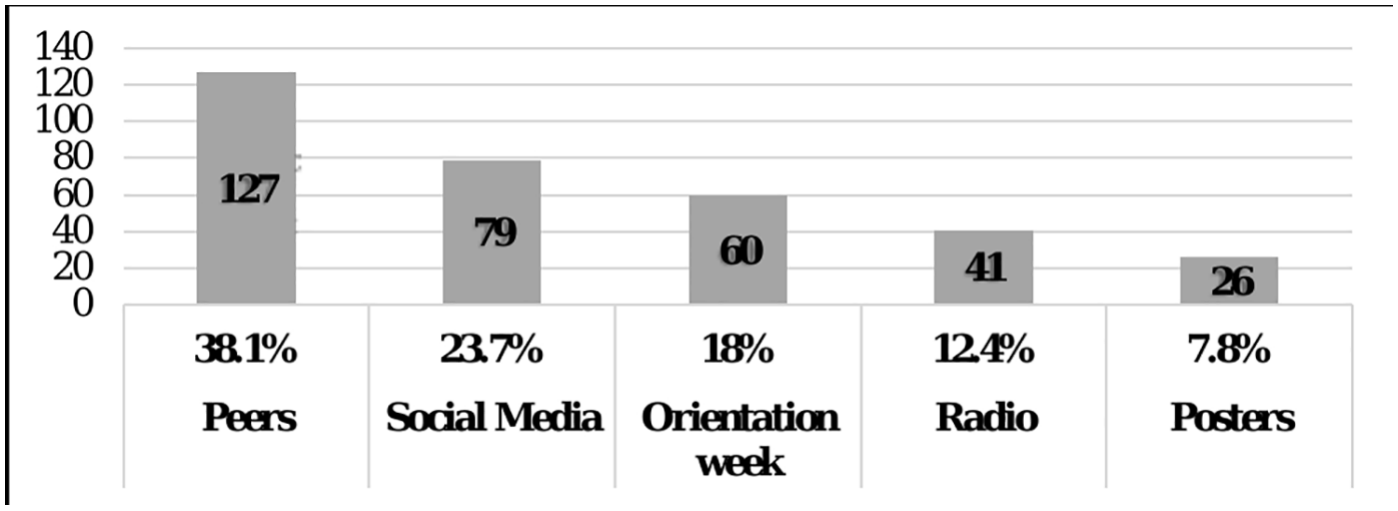
Sources of oral PrEP information affecting youth at MUBAS, Malawi.

In contrast, the majority of respondents (38.1%, n = 127) received their information about oral PrEP from peers; the least (7.8%, n = 26) mentioned posters as their information source (Fig 3).

## Discussion

Determinants of HIV testing and oral PrEP uptake among youth were assessed using the HBM as a guiding framework. Selected HBM-related factors were associated with the outcomes, with a broader range of constructs identified for oral PrEP uptake compared to HIV testing. While age, a non-modifiable factor, was associated with HIV testing, oral PrEP uptake was influenced by multiple HBM constructs, including perceived threat of HIV infection, knowledge of the partner’s HIV status, and knowing someone who uses PrEP. These findings suggest that the HBM may better capture the behavioral drivers for oral PrEP than HIV testing in this population. In addition, social media emerged as an important self-reported cue to action for both HIV testing and oral PrEP.

In this study, 27.2% of respondents had never undergone an HIV test in their lifetime, indicating suboptimal uptake of HIV testing. The observed HIV testing uptake of 72.8% falls below the UNAIDS 95-95-95 target, which aims for 95% of youth to know their HIV status [27]. This underscores the continued need to strengthen HIV testing campaigns that target youth. Many respondents expressed a willingness to use oral PrEP. However, despite high willingness, the actual uptake of oral PrEP was notably low (21.7%). The finding should be interpreted with caution, as the study did not assess clinical PrEP eligibility or detailed behavioral risk factors. Low uptake may therefore partly reflect limited eligibility within this university sample rather than low acceptability.

In line with previous literature from Malawi arguing that younger populations are less likely to undergo HIV testing [28,29], our study identified that older students (21–24 years) had higher odds be tested for HIV. Similar age-related patters have been reported in other SSA settings, suggesting that the association may be consistent across different contexts [30–32]. Barriers for younger individuals include limited access to health facilities, lack of trust in healthcare workers, and HIV-related stigma [33–35]. Our finding underscores the importance of designing HIV/AIDS interventions for younger, sexually active youth (aged 18–20 years), considering that the median age of sexual debut in this study was 18 years. Universities represent a strategic setting for reaching this population early. Strengthening health education within university environment can equip students with the knowledge and skills needed to make informed decision. Regular campus-based campaigns that promote awareness of available preventive services, such as HIV self-testing may help increase uptake. Integrating life-skills development programs into university curricula can support younger youth in maintaining their values, allowing them to make confident health decision. In the context of the HBM, age is a non-modifiable modifying factor that may influence perceptions of susceptibility and benefits, which in turn affect testing behavior.

The associations between quarterly testing and both HIV testing and PrEP uptake were expected. Quarterly testing is a routine component of HIV prevention guidelines and an integral part of safe PrEP use [20]. These findings may primarily reflect adherence to recommended protocols rather than independent behavioral factors. However, prior engagement with HIV testing services has also been linked to increased willingness to initiate PrEP in other settings [25,36–38]. Adolescents aged 10–19 years who had never been tested demonstrated lower awareness of PrEP availability [22]. Our findings highlight the need to strengthen integrated HIV preventive strategies. Expanding decentralized HIV testing approaches, such as university-based testing can improve accessibility and normalize regular testing among youth. Integrating HIV testing into routine student health services could further facilitate timely PrEP initiation. Strengthening the linkage between HIV testing, counseling and PrEP services may support sustained engagement across the HIV prevention cascade.

Gender, a key demographic modifying factor, was associated with oral PrEP uptake. Male respondents had higher odds of PrEP use, while female respondents, who are more than twice as likely to live with HIV [39], had lower odds. Similar disparities have been reported in other settings, with women having lower PrEP coverage than men [40,41]. These differences likely reflect underlying social and structural factors. In some contexts, men often have greater sexual freedom, while young women who are sexually active before marriage may be perceived as promiscuous, discouraging them from accessing PrEP [33,42]. Gender inequality and fear of judgement from family or community members can further limit PrEP use [43,17]. Age-disparate relationships and gender norms create power imbalances that limit adolescent girls and young women’s (AGYW) autonomy in decision-making [44]. These intersectional factors increase AGYW’s vulnerability to HIV infection and limit their ability to access preventive services. There is need for gender-sensitive interventions that address structural barriers and empower AGYW to access preventive services. Public awareness of gender disparities in uptake is crucial to encourage behaviors that promote use [41]. Strategies such as Malawi’s National Girls’ Education Strategy and the establishment of safe spaces for AGYW may help mitigate social and cultural barriers. Fostering peer engagement, and implementing life-skills and empowerment programs can support informed decision-making regarding sexual health and PrEP use.

Respondents’ perceived threat of HIV infection was associated with PrEP uptake, supporting the predictive assumptions of the HBM [18]. Consistent with previous research, perceived HIV risk has been identified as a key motivator for PrEP initiation among youth [25,43,45–48]. Evidence from Malawi similarly revealed that AGYW expressed greater interest in PrEP when their partners’ HIV status was unknown, reflecting heightened perceived severity and a desire for protection [42]. The consistency of our finding with studies from SSA indicates that perceived threat plays a broader role in PrEP uptake beyond the study population. Interventions that increase youths’ awareness of HIV- risk could enhance PrEP uptake. Targeted HIV risk education integrated into university health services, as well as digital campaigns aiming at the seriousness of HIV infection may increase perceived susceptibility and severity and motivate preventive behavior [12].

The perceived threat of HIV infection may also be shaped by knowledge of the partner’s HIV status. In our study, this knowledge emerged as a significant factor for oral PrEP uptake. This indicates the importance of partner-related dynamics in shaping HIV preventive behaviors among youth. However, the finding diverges from previous studies from South Africa and Uganda where PrEP use was higher among individuals unaware of their partner’s status [43,47,49,50]. Similarly, a study in Malawi found that AGYW who were unaware of their partners’ HIV status were more likely to initiate oral PrEP [42]. In those contexts, uncertainty appears to drive perceived risk and motivate preventive behaviors. In contrast, the pattern observed in our study suggests a different underlying mechanism. Youth who engage in partner communication may be more receptive to PrEP as part of a broader prevention strategy, rather than relying on uncertainty as a trigger for uptake [51]. The association may reflect a subgroup involved in more communicative and health-oriented relationships. The finding reveals the need to interpret partner-related factors within their specific social and epidemiological contexts. In settings where communication and disclosure are more normative and supported by interventions, knowledge of the partner’s HIV status may act as a cue to action that reinforces preventive behaviors. Prompting couple-based HIV testing and open communication could strengthen engagement across the HIV prevention cascade [52].

Consistent with existing literature, awareness about oral PrEP was associated with increased use [14,22,46]. A scoping review highlighted the positive influence of awareness on oral PrEP uptake [53]. Given that respondents were urban and educated, the extent to which these patterns reflect awareness and uptake in the broader youth population in Malawi, particularly out-of-school or rural youth, remains uncertain. Nevertheless, the consistency of our findings across multiple contexts suggests that awareness is an important determinant of PrEP uptake more generally. To translate awareness into action MoH and youth-focused organizations could integrate PrEP education into university-based health services as well as community outreach programs. Peer-led initiatives and digital campaigns could further reinforce knowledge of PrEP, supporting informed decision-making and improving uptake among diverse groups.

In our study, respondents who reported that they could imagine taking a daily oral PrEP pill had higher odds of actual oral PrEP uptake. The finding highlights the motivational factors, such as readiness and positive attitudes toward PrEP, are important determinants of initiation among youth. Consistent with other research on PrEP intentions, higher willingness has been linked to increased likelihood of use [22,42,54,55]. A qualitative study in Malawi found that AGYW who were informed about oral PrEP expressed interest in using it [42]. Youth education and evaluation of personal HIV risk may help increase willingness which in turn could support uptake. Promoting knowledge of PrEP’s effectiveness may be critical to help potential users make informed choices regarding prevention [42]. Understanding the relationship between willingness and actual uptake in HIV prevention programs should be prioritized in further PrEP implementation research. The extent to which willingness predicts actual uptake may vary depending individual circumstances and the context in which PrEP is provided.

Knowing someone taking oral PrEP was associated with higher odds for oral PrEP uptake, underscoring the importance of peer influence and personal connections in encouraging PrEP initiation [18]. Within the HBM framework, interpersonal experiences can serve as cues to action, motivating individuals to engage in preventive behaviors [56]. Evidence from HIV research indicates that knowing someone with HIV or AIDS positively influences preventive health behaviors [57]. A systematic review across multiple populations found that social support from partners or peers significantly boosted PrEP uptake [58]. Studies from Uganda, Zimbabwe, South Africa, and Kenya reported similar effects [10,47]. The finding suggests that peer influence observed in this university sample may be generalizable to other youth populations in SSA. In institutional settings *peer navigators* (trusted peers trained to support access and adherence) can help bridge the gap between healthcare providers and youth, translating motivation and awareness into actual PrEP use [59,60]. Incorporating peer-led interventions into university health programs may be an effective strategy.

In addition, overall, 24.9% of respondents accessed information on HIV testing and 38.1% on oral PrEP through peers. Peer networks are a common source of information and influence engagement with preventive behaviors. Youth often rely on their peers for reproductive health information, which can foster open discussions but also carries the risk of spreading misinformation due to limited knowledge [61]. Evidence from Kenya revealed that the effectiveness of peer engagement and peer navigators influencing an increase in HIV testing uptake among adolescents [62]. Peer mentors are crucial for PrEP adherence among AGYW by ensuring appointments, tracing defaulters, and linking them to health clinics [10]. To strengthen HIV prevention and care strategies in campus-based clinics, peer involvement initiatives should be integrated into existing programs [63]. Consistent with previous studies, our findings show that social connectedness increase oral PrEP [64,65]. Peer referral, a social network-based strategy that leverages trust between individuals, can promote access to HIV testing and oral PrEP while providing social support and encouraging positive health behavior. These findings may be generalizable to similar youth populations in SSA, where peer networks strongly influence reproductive health behaviors. However, contextual differences, such as cultural norms and program infrastructure, should be considered when applying these strategies elsewhere. Social media platforms were reported as another important information channel, with 27.9% of respondents obtaining HIV testing information and 23.7% receiving information on oral PrEP. These findings highlight the relevance of digital platforms as sources of HIV-related information among youth [17]. This is important in Malawi, where half of the population is below 18 years of age [66], suggesting that social media-based interventions could reach a substantial portion of the youth population. However, the findings should be interpreted within the context of university students, who typically have greater access to digital technologies and higher digital literacy than the broader youth population in Malawi. A qualitative study in South Africa argued that youth favored platforms such as Facebook, Twitter, and WhatsApp, for HIV education, mentioning improved privacy as a key factor [50]. Similarly, research in SSA highlights the effectiveness of digital platforms in increasing awareness of oral PrEP among adolescents [67,68], while other studies report preferences for television advertisements and WhatsApp messaging [14]. Our findings suggest that integrating social media into HIV prevention strategies, particularly within educational institutions, may be effective. Digital platforms can reach youth more efficiently than traditional media, providing opportunities for personalized and engaging communication [69,70]. Interventions could leverage digital media by incorporating interactive campaigns and interactive-text messaging to increase HIV testing and oral PrEP uptake. However, external validity may be limited by disparities in access to technology and digital literacy, particularly among non-student youth, and by the risk of spreading misinformation [61]. To mitigate these challenges within university-settings, campus-based health clinics linked to mobile health applications could serve as effective intervention points.

A combination of peer influence and digital media may serve as effective cues in health interventions. Peer-driven programs and referral strategies to enhance motivation for HIV preventive behaviors among youth could be supported and amplified through digital platforms. Preventive behavior could be demonstrated by peers under health professional supervision and shared via social media to reach larger proportion of young people. The MoH should leverage social media for health information and peer communication, ensuring reliable internet access and youth-friendly platforms that provide accurate information and counter misinformation. Integrating peer engagement into sexual behavior campaigns can further enhance intervention effectiveness.

Overall, the HBM provided a useful framework for organizing and interpreting selected determinants of HIV preventive behaviors. However, the model was only partially operationalized in this study. HBM construct were not associated with HIV testing, indicating that complementary theoretical frameworks may further enhance understanding of HIV testing and oral PrEP uptake among youth.

## Conclusion

Key modifiable determinants, such as perceived threat of HIV, awareness of oral PrEP, knowing someone who uses PrEP, and knowing a partner’s HIV status were associated with oral PrEP uptake, underscoring the role of individual perception and cues to action. In contrast, no HBM construct were significantly associated with HIV testing, suggesting that testing behaviors may be more strongly influenced by structural and contextual factors beyond individual perceptions. Given that respondents predominantly accessed information through digital platforms and social exposure, health communication strategies leveraging these channels may serve as effective cues to action, potentially improving knowledge and risk perception. Peer-led approaches, particularly those using social media, podcasts, and other digital platforms, may further enhance engagement by delivering relatable and accessible health information to youth. Overall, the findings reveal that the HBM may be more applicable to understanding PrEP uptake than HIV testing in this context. Further research should consider complementary theoretical frameworks that better capture the broader social and structural determinants of HIV prevention behaviors.

### Limitations of the study

This study has several limitations. The cross-sectional study design does not allow for the establishment of causal relationships between identified determinants and HIV testing or oral PrEP. There is no evidence of a temporal relationship, as exposure and outcome were assessed simultaneously. The study was conducted at a single university in Malawi, which may limit the generalizability of the findings to other settings. Social desirability bias may have been present in the respondents’ reports of sexual risk behaviors, HIV testing history, and oral PrEP uptake. This study did not include a risk assessment of the respondents prior to the questionnaire, therefore, not all respondents were at substantial risk of HIV infection. Consequently, the proportion of respondents who reported oral PrEP uptake may underestimate uptake among those at genuine risk.

Logistic regression was used to estimate associations, with results presented as odds ratios. For outcomes that are not rare, odds ratios may potentially overstate the strength of associations compared to prevalence ratios. Alternative approach, such as Poisson regression with robust standard errors, could provide more directly interpretable estimates. Some of the estimated associations where accompanied by wide confidence intervals, indicating limited precision. This may be due to imbalanced distributions of responses, which can result in unstable estimates in regression models. Therefore, these findings should be interpreted with caution.

## Supporting information

S1 File Original questionnaire used for data collection. (PDF)

S1 Data Anonymized dataset underlying the findings of this study. (XLS)

## Abbreviations

AGYW: Adolescent girls and young women
AIDS: Acquired Immune Deficiency Syndrome
AOR: Adjusted Odds Ratio
CI: Confidence Interval
COR: Crude Odds Ratio
HBM: Health Belief Model
HIV: Human Immunodeficiency Virus
KuHeS: Kamuzu University of Health and Sciences
MoH: Ministry of Health
MUBAS: Malawi Business and Applied Sciences
PrEP: Pre-Exposure Prophylaxis
QECH: Queen Elizabeth Central Hospital
SD: Standard Deviation
SSA: Sub-Saharan Africa
STI: Sexual Transmitted Infection
UN: United Nations
UTT: Universal Test and Treat
