# Supplementary material for: Determinants of HIV testing and oral PrEP uptake among youth at Malawi University of Business and Applied Sciences: A cross-sectional study employing the Health Belief Model
Source: PLOS Glob Public Health. 2026 Jun 24;6(6):e0006324. doi: 10.1371/journal.pgph.0006324 (PMC13293412; doi:10.1371/journal.pgph.0006324)
Supplement: S1 File — (PDF) [file pgph.0006324.s001.pdf]

QUESTIONNAIRE

PARTICIPANT IDENTIFICATION

Date____

ID Number____

SECTION A
SOCIO-DEMOGRAPHICS

N.	Questions	Answer	Options
**Q1**	Are you a male or female?	Male ☐	Female ☐
**Q2**	How old are you?	Age ☐☐	
**Q3**	What program are you pursuing at MUBAS?	Diploma ☐<br>Undergraduate ☐	Postgraduate ☐
**Q4**	What is your tribe or ethnic group?	______________________	
**Q5**	What is your marital status?	Single ☐<br>Married ☐<br>Single and dating ☐<br>Others specify<br>______________________	Divorced ☐<br>Casual dating ☐<br>Having a partner ☐
**Q6**	I have sex with	Men ☐<br>Both ☐	Women ☐<br>None ☐

SECTION B
ALCOHOL CONSUME

N.	Questions	Answer	Options
**Q7**	Do you take alcohol?	Yes ☐	No ☐
**Q8**	What is your alcohol quantity?	Occasional drinker ☐<br>Frequent drinker (daily) ☐	Moderate drinker (weekends) ☐<br>None ☐

SECTION C
SEXUAL ACTIVITY

N.	Questions	Answer	Options
**Q9**	How old were you when you had sexual intercourse for the very first time?	Age in years ☐☐	Never had sexual intercourse ☐
**Q10**	The last time you had sexual intercourse was a condom used?	Yes ☐	No ☐
**Q11**	How often do you use condoms during sex?	Always ☐<br>Never ☐	Sometimes ☐
**Q12**	How old is your partner?	Age of partner ☐☐	Don't know ☐
**Q13**	In total, with how many different persons have you had sexual intercourse in your lifetime?	Number of partners in lifetime ☐☐	Don't know ☐
**Q14**	Have you engaged in sexual intercourse without a condom at least once with each of your partners?	Yes ☐	No ☐

SECTION D
RISK PERCEPTION

N.	Questions	Answer	Options
**Q15**	Do you think you have a risk of getting HIV/AIDS or sexual transmitted infection?	Yes ☐	No ☐
**Q16**	Are you concerned about getting HIV/AIDS	Yes ☐<br>Not really ☐	Sometimes ☐

SECTION E
HIV TESTING

N.	Questions	Answer	Options
**Q17**	I don't want to know the result, but have you ever been tested for HIV?	Yes ☐	No ☐
**Q18**	How did you get the information about HIV testing?	Radio ☐<br>Friends ☐<br>Television ☐<br>Other specify<br>________________	Social Media ☐<br>Orientation week ☐<br>Poster ☐
**Q19**	How many months ago was your most recent HIV test?	Months ago ☐☐	Two or more years ☐

**Q20**	Do you test every three months?	Yes ☐	No ☐
**Q21**	Do you know your partner's HIV status?	Yes ☐	No ☐

SECTION E
PRE-EXPOSURE PROPHYLAXIS (PrEP)

No.	Questions	Answer	Options
**Q22**	Are you aware of PrEP as an HIV preventive measure?	Yes ☐	No ☐
**Q23**	How did you get the information about PrEP?	Radio ☐<br>Friends ☐<br>Orientation week ☐<br>Others specify<br>________________	Internet ☐<br>Posters ☐<br>Television ☐
**Q24**	Where did you get PrEP?	MUBAS Clinic ☐<br>Lighthouse ☐<br>I have never taken PrEP ☐	Queen Elizabeth Central Hospital ☐<br>Others specify<br>________________
**Q25**	Do you know someone taking PrEP?	Yes ☐	No ☐
**Q26**	Would you take one pill a day to prevent HIV?	Yes ☐	No ☐
